# Silsesquioxane-Doped Electrospun Nanofibrillar Membranes for Separation Systems

**DOI:** 10.3390/polym14173569

**Published:** 2022-08-30

**Authors:** Miłosz Frydrych, Bogna Sztorch, Dariusz Brząkalski, Rafał Kozera, Roksana Konieczna, Tomasz Osiecki, Robert E. Przekop

**Affiliations:** 1 Faculty of Chemistry, Adam Mickiewicz University in Poznań, 61-614 Poznań, Poland; 2 Centre for Advanced Technologies, Adam Mickiewicz University in Poznań, 61-614 Poznań, Poland; 3 Faculty of Material Science and Engineering, Warsaw University of Technology, 02-507 Warsaw, Poland; 4 Department of Mechanical Engineering, Technische Universität Chemnitz, Straße der Nationen 62, 09111 Chemnitz, Germany

**Keywords:** electrospun, silsesquioxane, nanofiber, polylactide, PLA, membrane

## Abstract

In this study, a series of cage siloxanes (CS), e.g., three polyhedral oligomeric silsesquioxanes (SSQs) and one spherosilicate (SS) derivative, were applied as functional additives for the preparation of poly(lactic acid)-based (PLA) nanofibrillar membranes with an electrospinning technique utilizing an efficient spinning wire electrode setup. The impact of the additives’ structure, chemistry, and electrospinning parameters on the obtained materials’ morphology (scanning electron microscopy) and physicochemical (thermogravimetry, differential scanning calorimetry, contact angle analysis, air flow analysis) properties is discussed. It is presented that applying organosilicon additives may extend the already tuneable properties of the membranes produced by electrospinning performed under different conditions and that they enable to obtain nanofibres of smaller diameter, which in turn increases the membrane porosity. Furthermore, the solvent-assisted electrospinning method allowed for unparalleled mixing of the PLA matrix with the CS additives, as no traces of free additives were visible on the membranes by scanning electron microscopy (SEM) imaging. The resulting membranes can be utilized as filter materials.

## 1. Introduction

Electrospinning is a widely-used technique for creating polymer fibres using electrostatic forces [1]. This process enables the production of nanofibers and fabrics with controlled pore structure [2]. Of the many electrospinning techniques described in the literature, the Nanospider™ spinning technology deserves special attention, as it allows continuous and high-volume production of fabrics [3]. The main difference between the described method and classic sputtering is a rotating electrode in the form of a wire-surrounded bar, immersed in a polymer solution. The advantages of its use are high efficiency with its uniform electrostatic field, as well as its prevention of undesirable changes occurring in the solution during prolonged exposure [4]. The traditional needle method for the preparation of nanofibers does not provide sufficient amounts of material per unit of time, which either hinders preparation of enough sample mass/area for a number of different analytical methods for proper basic characterization of the material obtained, or runs a high throughput screening of different additives and loading fractions thereof [5,6]. Thanks to the unique properties of the obtained membranes, electrospinning has found wide applications in many fields, e.g.,

- For filtration systems, as separators of particles from suspensions and air [7,8,9,10];
- Tissue engineering, as implants and grafts for accelerated tissue regeneration for reconstructive surgeries [11,12,13,14,15];
- Biomedical materials [16], as novel dressings for burn victims (traumatology), bedsore patients, and other cases of necrosis-affected patients [17,18,19,20];
- Drug carriers [21,22];
- Cell culture growth matrices [23];
- Composite systems [24,25].

One of the most promising groups of compounds used as modifiers for plastics and polymers [26] are polyhedral oligomeric silsesquioxanes of the general formula [RSiO_1.5_]_n_ (where R can be a hydrogen atom, an alkyl group, an aryl group, and their derivatives, including heteroatom-bearing structures). Organofunctional silsesquioxanes are hybrid compounds that combine the features of inorganic and organic systems [27,28]. These compounds owe their popularity to an increasing number of applications in various industries, mainly due to good thermal and mechanical properties and the ease of their further functionalization via many processes of modern organic synthesis. Silsesquioxanes can be used as functional additives in the processing of thermoplastic and thermosetting polymers, as well as the production of composite materials (significantly improving their thermal and mechanical properties) in optoelectronics, microelectronics, pharmacy (drug carriers), medicine, and dentistry [29]. The most popular and widely studied group of silsesquioxanes are cage-shaped octasubstituted cubic silsesquioxanes. They are obtained from the hydrolytic condensation reaction of trichloro- or trialkoxysilanes [30,31,32], and further functionalized mostly by nucleophilic substitution, condensation, and addition, or catalytic reactions such as hydrosilylation in the presence of transition metal complexes [33]. Cage silsesquioxanes, due to their rigid molecular structure and nanometric dimensions, are characterized by strong dispersion properties, which can be tuned by the means of proper selection of functional substituents attached to the inorganic siloxane core.

Cage siloxanes (CS), in a form of either polyhedral silsesquioxanes (SSQ) or spherosilicates (SS), have been a subject of study of electrospinning process applications. The materials obtained by this process have a very thorough dispersion of spherosilicates at both the nano- and micro-scale [28], which determines their potential use in highly advanced materials. For example, fabricated membranes can have possible application in lithium-ion batteries. The addition of silsesquioxanes in a polymer matrix improved the electrochemical and mechanical properties of the composites. Fire retardancy tests confirmed strong thermal resistance of the obtained material. Assembled batteries had good cyclic performance and rated capacity [25,26,27,28,29,30,31,32,33,34,35,36,37,38]. Biomedical application of SSQs have been also reported [39]. SSQ functionalized with a polypeptide chain was combined with PCL to obtain membranes for antibacterial application. Results showed that obtained material had notable activities against *E. Coli* and *S. Aureus* [40].

In this study, the electrospinning technique was used to obtain polylactide (PLA) membranes doped with organosilicon modifiers comprising a group of cage siloxanes: functionalized silsesquioxanes (SSQs) and a spherosilicate (SS), the last one being studied earlier as an effective processing additive for PLA [41]. PLA is known for being a biofriendly, biodegradable, and bioresorbable material derived from the polymerization of naturally produced lactic acid (obtained in a fermentation process), characterized by partial transparency, moderately good mechanical properties and, depending on production conditions, amorphous or semi-crystalline structure. Herein, we present the findings on an approach to the preparation of PLA-based membranes modified with functionalized silsesquioxanes, that is, Hepta(isobutyl)trisilanolheptasilsesquioxane (Isobutyltrisilanol POSS^®^, iBu_7_SSQ-3OH), Ethoxyheptaisobutyloctasilsesquioxane (iBu_7_SSQ-OEt), Octakis(3-chloropropyl)octasilsesquioxane (SSQ-8Cl), and a limonene-derived octaspherosilicate (SS-Limonene) obtained by the electrospinning process.

## 2. Materials and Methods

### 2.1. Materials

The chemicals were purchased from the following sources: isobutyltrimethoxysilane, 3-chloropropyltriethoxysilane, and tetraethoxysilane from ABCR (Karlsruhe, Germany); chloroform-d from Sigma Aldrich (Poznan, Poland); phosphorus pentoxide, tetrahydrofuran (THF), toluene, methanol, hydrochloric acid, acetonitrile, and acetone from Avantor Performance Materials (Gliwice, Poland). PLA, Ingeo™ 2003D, was obtained from Natureworks LLC (Plymouth, MN, USA).

### 2.2. Organosilicon Precursors (Spherosilicates and Silsesquioxanes)

Limonene-substituted spherosilicate (SS-Limomene) was prepared according to the previous report [41]. Silsesquioxane additives were prepared according to literature reports: hepta(isobutyl)trisilanolheptasilsesquioxane (iBu_7_SSQ-3OH) [42], octakis(3-chloropropyl)octasilsesquioxane (SSQ-8Cl) [43], and Ethoxyheptaisobutyloctasilsesquioxane (iBu_7_SSQ-OEt) [44]. All the obtained compounds were subjected to NMR and FT-IR spectroscopy and the results were compared with the literature data to confirm their structure and purity. The compounds’ structures are provided in Table 1.

### 2.3. Preparation of the PLA Solution for the Electrospinning Process

In order to prepare the PLA stock solution, 83 g of PLA was dissolved in 1000 mL of chloroform by constant mechanical stirring over 24 h. Next, to obtain the working solution, 40 mL of stock solution was diluted with 40 mL of chloroform. To the obtained working solution, a chosen modifier in an amount of 5 wt% in accordance with the PLA present in the solution was added, and the solution was shaken until fully homogeneous.

### 2.4. Process Conditions

The membranes were obtained in an electrospinning process using an Elmarco NS LAB M Nanospider machine, comprised of an electrospinning chamber, a high-voltage power supply, an electrospinning power lead, a grounding lead, an electrospinning lead drive, and a fabric support for collecting the produced membranes. The process was performed under various conditions at room temperature. For each of the compositions prepared, four sets of parameters were tested, as collected in Table 2.

### 2.5. Analytical Methods

The obtained membranes were characterized by water contact angle analysis, thermogravimetric analysis (TG), and differential scanning calorimetry (DSC), and surface imaging was performed with Scanning Electron Microscopy (SEM).

Contact angle analyses were performed by the Sessile Drop Technique at room temperature and atmospheric pressure with a Krüss DSA100 goniometer. Three independent measurements were performed for each sample, each with a 5 µL water drop, and the obtained results were averaged, which was done to reduce the effects of surface nonuniformity.

Thermogravimetric analysis was performed using a NETZSCH 209 F1 Libra gravimetric analyser. Samples of 5 ± 0.2 mg were cut from each membrane and placed in Al_2_O_3_ crucibles. Measurements were conducted under nitrogen flow of 20 mL/min in the temperature range of 30÷750 °C and a 20 °C/min heating rate.

Differential Scanning Calorimetry was performed using a NETZSCH 204 F1 Phoenix calorimeter. Samples of 5.0 ± 0.2 mg were cut from each membrane and placed in aluminium crucibles with pierced lids. Measurements were done under a nitrogen flow in 20–220 °C temperature range and at 10 °C/min heating rate.

Scanning Electron Microscopy was performed using a Hitachi TM3000 Tabletop Microscope equipped with an SDD detector. The images were captured in a 100–4000 magnification range.

Gas permeation measurements were performed on an individually designed setup, consisting of a membrane vacuum pump, a 200 L steel vacuum tank, an electronic vacuometer, a set of manual ball valves, and a sample holder attached to the vacuum tank, comprised of two steel rings, a steel clamp, and a flange connection. The measurement involved placing a sample of the membrane-coated fabric substrate (the fabric substrate being highly porous so its presence alone would have a negligible impact on the gas flow), as obtained from the electrospinning process between the rings of the sample holder; securing the rings with the clamp; reducing the pressure in the vacuum tank to 700 mbar with the vacuum pump; closing the valve connecting the vacuum tank and the vacuum pump to stabilize the pressure in the tank; opening the valve connecting the vacuum tank with the sample holder; and measuring the pressure change over time with the digital vacuometer.

## 3. Results

### 3.1. Scanning Electron Microscopy

**Figure 1:**
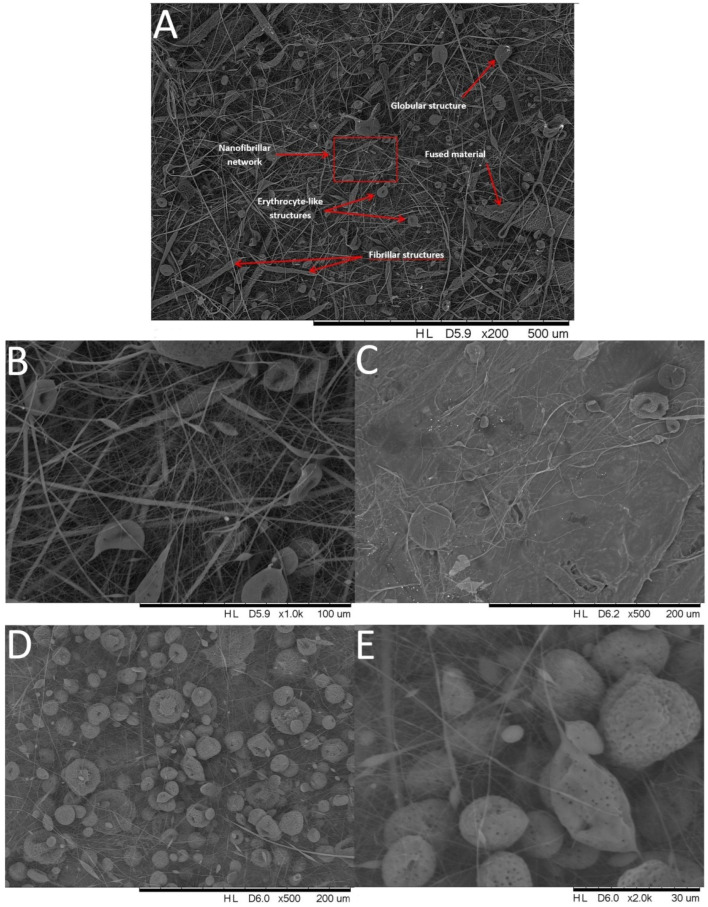
SEM images of the neat PLA electrospun membranes: (**A**) Membrane obtained under conditions (2) with different structures formed being shown; (**B**) A red rectangle section from image (**A**) under higher magnification; (**C**) Foil-like structure obtained under conditions (3); (**D**) Membrane obtained under conditions (4); (**E**) A section from image (**D**) under higher magnification.

**Figure 2:**
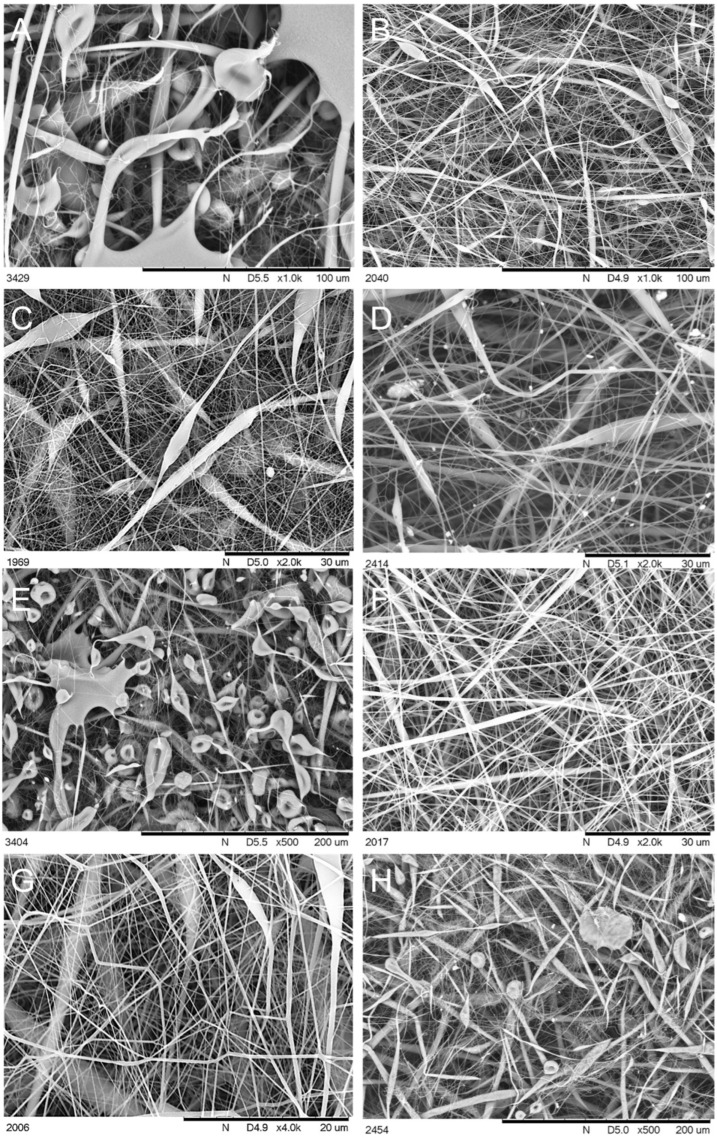
SEM images of membranes electrospun from PLA doped with cage siloxane compounds used in this study; (**A**) S-Limonene under condition (2); (**B**) SS-Limonene under condition (3); (**C**) iBu_7_SSQ-3OH under condition (2); (**D**) iBu_7_SSQ-3OH under condition (3); (**E**) iBu_7_SSQ-OEt under condition (1); (**F**) iBu_7_SSQ-OEt under condition (2); (**G**) SSQ-8Cl under condition (2); (**H**) SSQ-8Cl under condition (3).

Scanning Electron Microscopy (SEM) was performed to analyse the morphology of the obtained materials. It was observed that for most systems, conditions 2 and 3, which utilized a lower electrode spin velocity, produced fibres of more uniform shapes and lower diameters, as well as lower formation of regions of amorphous, fused material or droplet-shaped microglobules. Moreover, the increased voltage applied under condition (2) produced more uniform shapes of fibres and reduced the number of observable defects, as well as allowed for obtaining the highest yield of nanofibers. In general, three types of structures were observed, besides the completely fused regions (Figure 1): the fibril structures of large diameter (several micrometres) and low aspect ratio (<30); nanofibers of diameter <1 µm and large aspect ratio (>30, up to hundreds, Figure 1b); and microglobular, droplet-like structures mentioned earlier. Some of these had a cavity, giving them a very characteristic erythrocyte-like shape. For the neat PLA, all of these structures were observed, but under condition (1), no formation of fused regions was observed, and under condition (2), those were rare and occupied a close-to-negligible fraction of sample surface. Condition (3) yielded heavily fused, foil-like material with other structures being deeply embedded in it (Figure 1c), and condition (4) produced fused material with more porosity and large amounts of highly porous globules (Figure 1d,e), a structure not observed for any other of the samples discussed further. Interestingly, when inspecting the CS-doped membranes, there was no sign of the additives’ crystallites within the material, showing effective mixing of the additives with the matrix under the solvent-assisted electrospinning procedure (Figure 2). For SS-Limonene, condition (2) was the only one to produce a satisfactory yield of nanofibers, while the other systems varied in the amounts of fused material produced. Under (2), high yields of fibres ~1 μm in diameter were observed, with significant amounts of nanofibers being below 500 nm. The other samples of the SS-Limonene/PLA system were abundant with erythrocyte-like, discus-shaped microglobules ranging in diameter from ~10 to 40 μm. iBu_7_SSQ-3OH/PLA showed severe fusion under (4) and some under (1), resulting in highly porous structures with both foil-like and globular structures, while conditions (2) and (3) allowed for the successful formation of fibrillar networks. The two latter systems presented a characteristic feature of fibrils of several hundreds of micrometres long and up to 10 μm in diameter, the length/diameter ratio being significantly smaller than that of the corresponding nanofibers. The nanofibers in (2) were mostly of <300 nm in diameter. For iBu_7_SSQ-OEt, fusion was less severe than for the two abovementioned compositions and was visible under conditions (1) and (4) to a limited extent with numerous erythrocyte-shaped structures visible, constituting the most abundant motifs in the image. Fibril structures were also visible; however, fibres and nanofibers constituted a larger fraction of the material volume. The nanofibers under (2) were mostly of ~500 nm in diameter. The composition of SSQ-8Cl showed the least fusion; however, the systems were abundant in globular and fibril structures, especially (4) and (3). For (2), the nanofibers were mostly of 300–500 nm in diameter.

### 3.2. Thermal Analysis

#### 3.2.1. TGA Analysis

**Figure 3:**
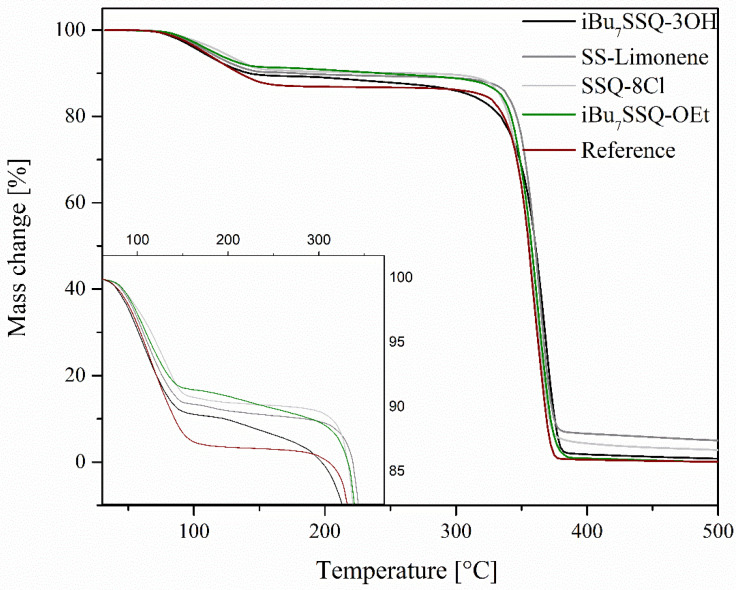
TGA curves of the obtained electrospun materials.

**Figure 4:**
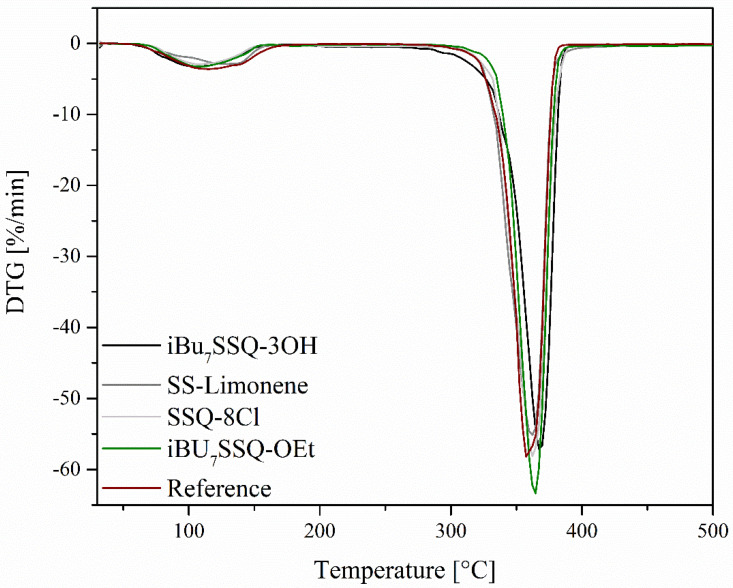
DTG curves of the obtained electrospun materials.

**Figure 5:**
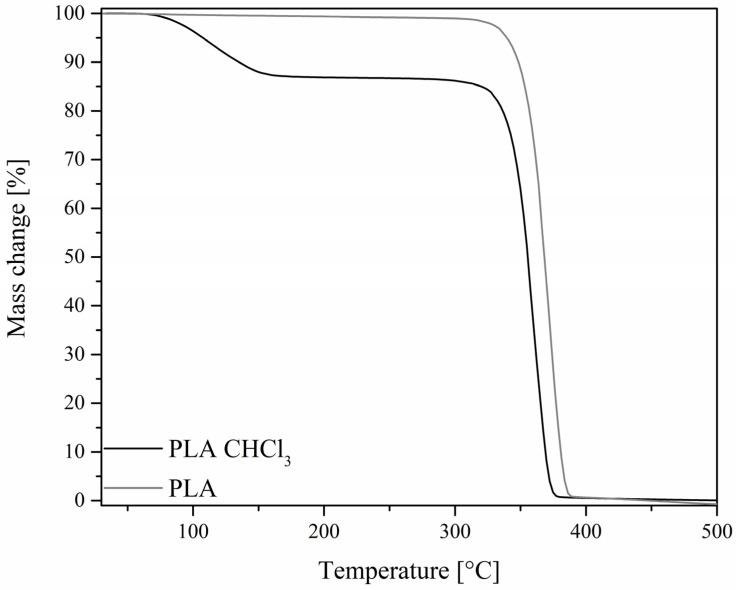
TGA curves of hot-pressed and solvent-cast PLA foils.

On the basis of the obtained TGA curves (Figure 3 and Figure 4), T_5%_, T_onset,_ and T_max_ of both degradation events have been determined, as well as the mass loss after the first degradation event and the total mass loss (Table 3). The TGA curves show that the obtained membranes undergo initial mass loss in the low temperature range of 60–150 °C. This is much lower than what is usually reported for bulk PLA [45]. It was speculated that the effect was due to either residual chloroform remains absorbed by the polymer, evaporating at elevated temperatures, or the material micro- and nanostructure, giving the high porosity of the membrane increasing surface area subjected to reaction with the oxygen. Naeem reported unusually low initial mass loss (starting at around 80 °C) when preparing PLA composite films, TGA curves presenting similar patterns to those of the membranes presented in this work [46]. The preparation of the samples was similar, i.e., from PLA solutions in chloroform. To better evaluate the speculated solvent effect, two additional samples of similar thickness were prepared in a film form, one being cast from PLA solution in chloroform and one by hot pressing, and their TGA curves were compared (see Figure 5). In this case, the initial low-temperature mass loss visible for a cast film and virtually identical with results obtained for electrospun samples, is absent from a hot-pressed sample, which proves that this mass loss can be attributed to chloroform evaporation. The temperature range of this event (60–150 °C), beginning around the boiling point of chloroform but strongly exceeding it upon completion, can be explained by the low freedom of polymer chains, PLA being in a glassy state in this temperature range and reaching melting temperature at around 150 °C, which enables the full elimination of CHCl_3_ only at this point [46].

It is evident that the reference PLA membrane shows the highest initial mass loss, which corresponds to the highest amount of residual chloroform in the sample. This is due to the molecules of the organosilicon additives taking up the polymer’s free volume, leaving less volume for the solvent. This correlates with the molecule size, as SSQ-8Cl and iBu_7_SSQ-OEt samples show the least amount of solvent absorbed, while small molecules of additives take up the free volume the most effectively. Silsesquioxane trisilanols are known for existing in a dimeric form in aprotic environments or a solid state [47,48], and SS-Limonene is a large molecule due to the spherosilicate corona and large, cycloaliphatic substituents. Also, different temperatures of maximum mass loss at the first stage, measured by DTG peak (T_max1_), show the interaction between additive molecules and polymer chains, iBu_7_SSQ-3OH, SSQ-8Cl and iBu_7_SSQ-OEt increasing chain mobility and therefore accelerating solvent thermal elimination, while SS-Limonene strongly interacts with PLA chains, reducing chain mobility.

At higher temperatures, when the main degradation event occurs, all the samples present similar behaviour; however, a slightly stabilizing effect may be noted, mostly for iBu_7_SSQ-3OH, where a 13 °C rise of the DTG peak was observed. Taking into account the different mechanisms of PLA thermal oxidative degradation [49] and the measured DTG peaks for maximum degradation rate, these suggest that the organosilicon additives either moderate undergoing free-radical reactions or they react with some of the degradation intermediates, forming non-volatile products. This observation presents the potential application of iBu_7_SSQ-3OH as a thermal degradation inhibitor for PLA processing or application in oxidative environments.

#### 3.2.2. DSC Analysis

**Figure 6:**
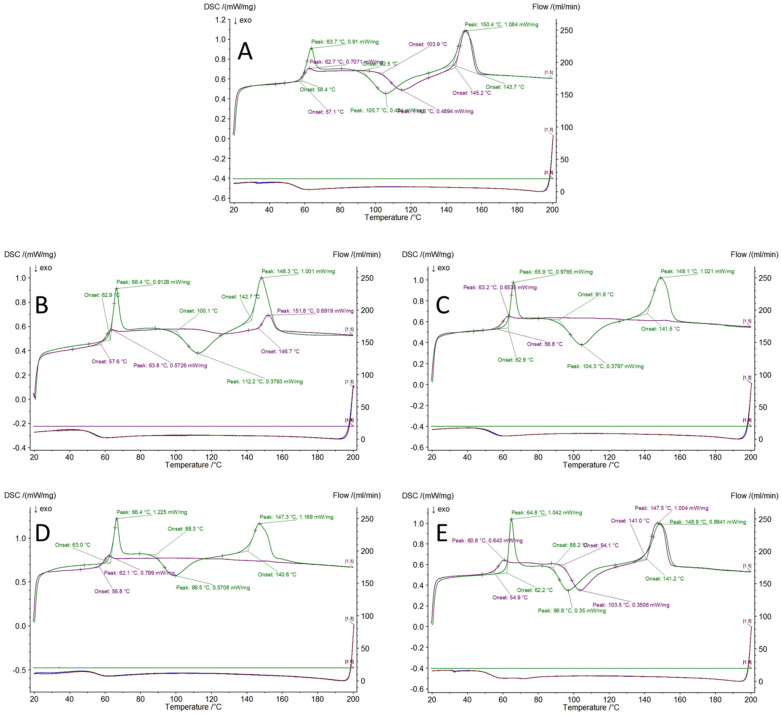
DSC curves of the first (green line) and second (violet line) heating of the electrospun membrane materials; (**A**) Neat PLA membrane; (**B**) PLA/SS-Limonene; (**C**) PLA/iBu_7_SSQ-3OH; (**D**) PLA/iBu_7_SSQ-OEt; (**E**) PLA/SSQ-8Cl.

Differential Scanning Calorimetry allowed us to observe the effects of doped PLA microstructure and organosilicon compounds on the thermal behaviour of the obtained materials. Interestingly, there was little to no difference between the samples of a given composition produced under different conditions, proving that the most important factors affecting the thermal behaviour are the choice of the CS compound and the processing method giving PLA its micro- and nanostructural characteristics; however, the quantitative effect of the particular conditions selected for the membrane fabrication are the least meaningful factor, as all of the materials are structured to exhibit micro- and/or nanofeatures. Therefore, the presented data for all the samples will be given for the samples obtained under conditions set (2) (Figure 6). A larger T_g_ endotherm is visible during the first heating cycle than for the second one, which can be caused by the high surface area of the samples, therefore exposing a large polymer phase and exhibiting more freedom (chain mobility) than that of the bulk polymer. This effect has been known and studied in detail for various polymers [50]. This large surface area is then destroyed due to polymer melting, which causes a reduction of the heat of this endotherm on the second heating cycle. An additional cause of the higher endotherm heat is the result of residual chloroform evaporation, in which matching the onset of glass transition and confirming that polymer relaxation causes the escape of the polymer from the void volumes it occupied under solid state of the polymer matrix. The effect of further solvent evaporation is further visible as a slight bulging of the DSC curve between the T_g_ and T_cc_ events. The high surface area and residual solvent presence are two reasons for the increased chain mobility in the polymer phase, which resulted in a heavy shift of the T_cc_ event from ~104 °C for the second cycle to ~92 °C for the first one, and a similar shift in the peaks of these events. The melting event, however, occurred in an almost identical manner, considering the onset temp, peak temp, and process enthalpy, as at this point the sample has no residual solvent and the cold crystallization event may be seen as an at least partial removal of the polymer processing memory. In the case of the CS-doped membranes, it is crucial to understand the thermal transitions of the organosilicon additives as well. For SS-Limonene, the additive was proven to undergo thermosetting polymerization above 110 °C with the exothermic peak at 162 °C when exposed to heat as a neat compound [26]. The effect of the additive polymerization is visible as a slight bulge of the DSC curve after the melting event during the first heating. The effect of the additive thermosetting might have caused the restraint of the PLA chain mobility, preventing the cold crystallization event and therefore the melting enthalpy observed. Additionally, due to the polymer void volume being partially occupied by the additive, the T_g_ onset is significantly higher for PLA/SS-Limonene than for the neat PLA (>5 °C). The same effect was visible for the remaining materials that were doped with silsesquioxanes. iBu_7_SSQ-3OH was also previously proven to undergo melting and thermal condensation with the formation of a complex mixture of products [51]. No crystallinity was observed for iBu_7_SSQ-OEt, either. These compounds seemed ineffective in providing nucleation to PLA and possibly also imparted some chain mobility restraint, which resulted in a completely non-crystalline structure of the polymer on the second heating, which might also be due to polymer chain restraints induced by iBu_7_SSQ-OEt or iBu_7_SSQ-3OH and its condensation products, all these compounds being potentially reactive towards grafting onto PLA chains in polymer melt temperature. SSQ-8Cl, being the only liquid and non-reactive CS compound studied in this work, provided reduction of the T_g_ onset on the second heating, showing its plastifying effect on the PLA matrix while not disturbing the cold crystallization or melting events.

### 3.3. Contact Angle Analysis

Water contact angle analysis was performed to study the membranes’ surface hydrophobic–hydrophilic properties. Most of the obtained systems exhibited quite similar values falling between 130–140°, classifying them as hydrophobic materials (Table 4). This value is larger than for neat, solid PLA, which is <90° and therefore hydrophilic, proving that electrospinning allows for structuring hydrophilic PLA into hydrophobic nanomaterials [41]. The addition of CS agents resulted in an increase of these values in most systems’ cases, with SS-Limonene being an exception due to the material fusion effect (see Section 3.1). iBu_7_SSQ-3OH and iBu_7_SSQ-OEt showed very similar impacts on the hydrophobic properties of PLA due to chemical similarity, while SSQ-8Cl was slightly less effective. From the point of view of filtering water-based media, it might be advantageous to use hydrophobic material due to less saturation and therefore limiting the water sorption and swelling by the membrane material.

### 3.4. Membrane Air Flow Analysis

To evaluate the viability of the membranes’ application for filtration systems, the flow resistance was measured for all the studied systems suitable for the test. The materials that turned out either too fragile or fused into non-porous foils were omitted in this test. Some general conclusions may be drawn, thus allowing for finding a linkage between the microstructure and permeability of the produced materials. The main aspects of the microstructure–permeability relationship are:

- The main diameter of the fibres, the nanofibers providing higher flow resistance than large fibrils;
- Formation of microglobules instead of fibres, resulting in lower packing density and lower flow resistance;
- Percolation effect between fibrillar and globular structures, increasing packing density and flow resistance;
- Formation of fused material regions, blocking the gas flow in the affected region.

**Figure 7:**
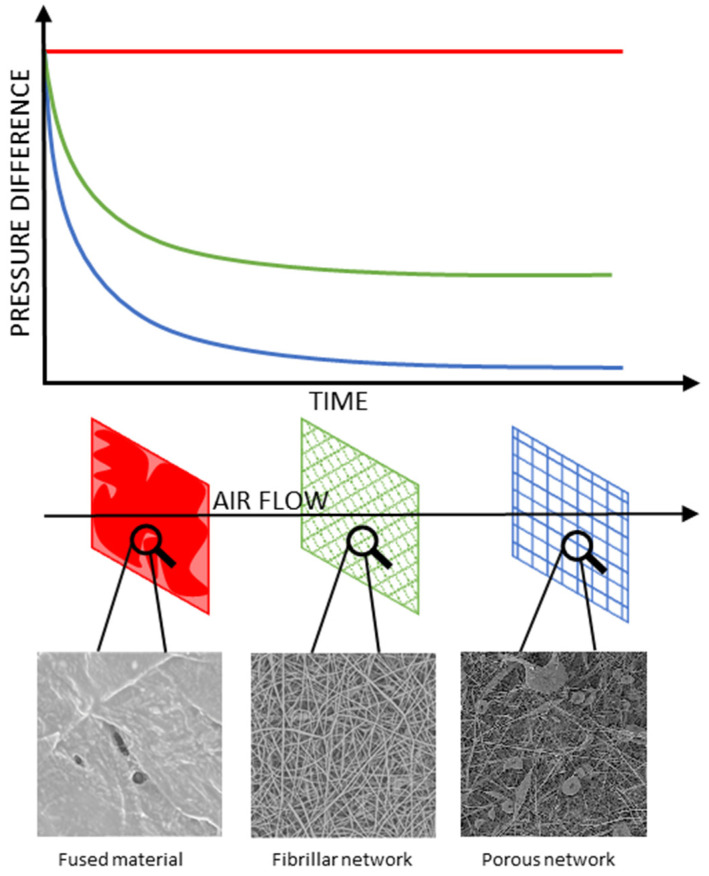
The microstructure–flow permeability relationship for the studied electrospun membranes.

**Figure 8:**
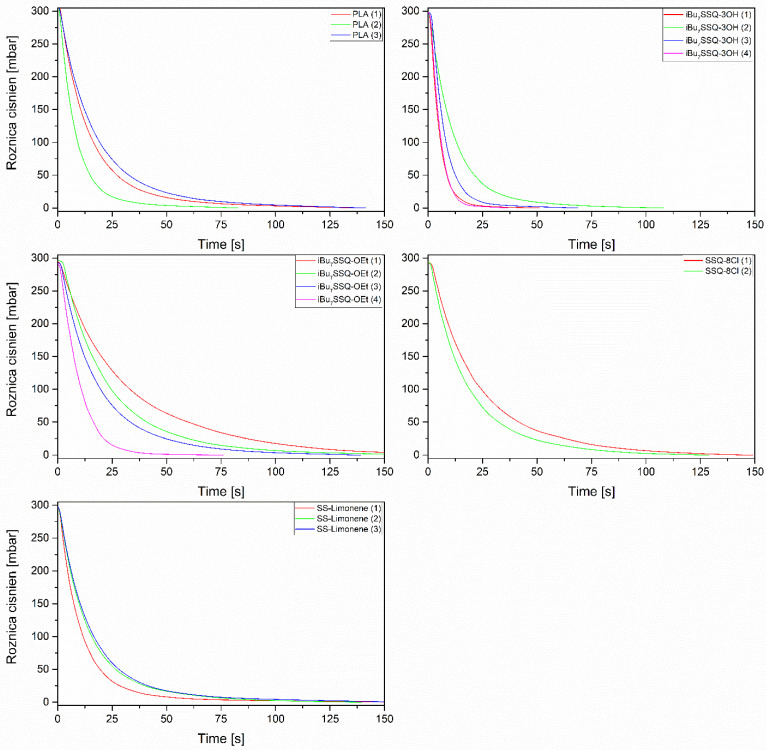
Flow resistance curves of the electrospun membranes prepared in this work.

These observations are presented in a simplified manner in Figure 7. The flow resistance curves obtained for the electrospun membranes are connected on Figure 8.

For neat PLA, SSQ-8Cl/PLA and SS Limonene/PLA under condition (4), the materials were fused into amorphous, foil-like brittle structures and could not be measured. As can be seen, only iBu_7_SSQ-3OH/PLA and iBu_7_SSQ-OEt/PLA allowed for the fabrication of membranes under all applied condition sets (1)–(4), proving the effect of the selected silsesquioxanes as a processing aid for fabrication of the electrospun fibres. On the other hand, SSQ-8Cl/PLA produced material too fragile to manipulate when processed under condition (3).

For iBu_7_SSQ-3OH/PLA, both condition sets 1 and 4 resulted in quite similar gas permeation kinetics due to high morphological similarity between the samples produced, that is, significant amounts of fused material connected by a sparse fibrillar network containing numerous globular inclusions. These fused regions do not participate in the gas permeation process, as they completely block gas flow and may only account for gas diffusion, which is a significantly slower process. On the other hand, conditions (2) and (3) allowed for obtaining high yields of fibrillar networks, which resulted in higher permeation resistances. Sample (2) provided higher resistance due to a larger volume of nanofibres present in the materials, as visible in the SEM images.

For iBu_7_SSQ-OEt/PLA systems, conditions (2) and (3) produced similar samples and, for iBu_7_SSQ-3OH/PLA alone, the lower permeation kinetics may be linked to the higher yield of nanofibres produced. For (1) and (4), some material fusing is visible, together with discus-shaped microglobules resembling erythrocytes. The main morphological differences accounting for drastically lower kinetics for system (1) is porous-like morphology, where a percolation effect occurred between nanocomposite microglobules, forming a highly porous network of high rigidity.

For SSQ-8Cl/PLA, the lower permeability of system (2) over (3) was due to a higher nanofiber/microglobule ratio, resulting in a denser fibrillar network.

For SS-Limonene/PLA, increased permeability of (1) was due to an increased yield of globular structures present.

Depending on the viscosity of the filtered medium and on the particle size distribution of the material to be filtered out, a different membrane characterized by its porosity and permeability may be considered as an optimal system of choice.

## 4. Conclusions

PLA-based, cage siloxane-doped membranes consisting of nanofibrillar networks have been prepared by an electrospinning method utilizing rotary wire electrodes for dispensing the polymer solution. The application of spinning wire electrodes allowed for fast fabrication of the membranes, while the solvent-assisted method provided unparalleled mixing of cage siloxanes within the PLA matrix. The electrospun membranes are hydrophobic, which is a structurization effect, while the application of silsesquioxanes enhances its magnitude. The organosilicon compounds, especially SSQ-8Cl and iBu_7_SSQ-3OH, allowed for producing nanofibrillar networks consisting of larger amounts of nanofibers of smaller diameters than the neat PLA. The CS additives occupied the polymer void volume, as suggested by the thermal analysis data. Chloroform also occupied this volume and remained trapped in the polymer until heated to PLA glass transition temperature, which may require elimination if such membrane systems were meant for applications such as water purification. All of the observed effects prove that the applied silsesquioxane and spherosilicate additives may extend the already tuneable properties of the membranes produced by electrospinning performed under different conditions. Based on the characteristics of the medium to be filtered and the properties of the particles suspended within to be filtered out (particle size distribution), different membranes characterized by their porosity and permeability may be considered as an optimal system of choice.

## Figures and Tables

**Table 1:** Structure and codes of the modifiers used in this study.

Code	Structure
iBu_7_SSQ-3OH	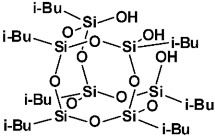
SS-Limonene	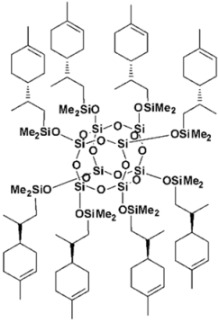
SSQ-8Cl	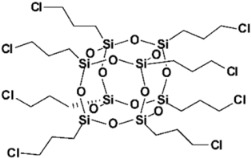
iBu_7_SSQ-OEt	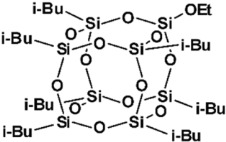

**Table 2:** Process parameters for electrospinning of PLA membranes.

Conditions Number	Voltage [kV]	Electrospinning Lead Rotation [rpm]	Time [min]
1	80	24	10
2	80	12	20
3	54	12	20
4	54	24	10

**Table 3:** Data collected from TGA and DTG curves.

	T_5%_ [°C]	T_onset_ [°C] ^[a]^	1st Stage		2nd Stage	Total Mass Loss [%]
			T_max1_ [°C]	Mass Loss [%]	T_max2_ [°C]	
iBu_7_SSQ-3OH	104.7	350.7	105.8	10.75	372.5	100
iBu_7_SSQ-OEt	114.6	348.8	109.3	9.75	364.2	96.63
SSQ-8Cl	119.7	344.5	109.4	8.64	359.5	100
SS-Limonene	109.6	340.9	120.5	9.23	362.1	98.53
Reference	107.3	352.5	114.9	12.90	359.4	100

^a^—the onset temperature provided for the material degradation event.

**Table 4:** Water contact angles of the prepared membranes.

System	Contact Angle Value
iBu_7_SSQ-3OH (1)	138.0
iBu_7_SSQ-3OH (2)	140.1
iBu_7_SSQ-3OH (3)	144.0
iBu_7_SSQ-3OH (4)	137.0
SSQ-8Cl (1)	132.9
SSQ-8Cl (2)	136.8
SSQ-8Cl (3)	136.0
SSQ-8Cl (4)	129.4
iBu_7_SSQ-OEt (1)	138.4
iBu_7_SSQ-OEt (2)	131.5
iBu_7_SSQ-OEt (3)	140.2
iBu_7_SSQ-OEt (4)	139.8
SS-Limonene (1)	137.3
SS-Limonene (2)	140.2
SS-Limonene (3)	129.3
SS-Limonene (4)	124.7
Reference (1)	129.9
Reference (2)	130.2
Reference (3)	129.9
Reference (4)	130.6

## Data Availability

All the data produced has been provided directly in the manuscript.

## References

[1] Bhardwaj N., Kundu S.C. (2010). Electrospinning: A Fascinating Fiber Fabrication Technique. Biotechnol. Adv..

[2] He J.-H., Wan Y.-Q., Yu J.-Y. (2005). Scaling Law in Electrospinning: Relationship between Electric Current and Solution Flow Rate. Polymer.

[3] Jirsak O., Sanetrnik F., Lukas D., Kotek V., Martinova L., Chaloupek J. (2004). Method of Nanofibres Production from a Polymer Solution Using Electrostatic Spinning and a Device for Carrying out the Method. U.S. Patent.

[4] Yalcinkaya F. (2019). Preparation of Various Nanofiber Layers Using Wire Electrospinning System. Arab. J. Chem..

[5] Rożek Z., Kaczorowski W., Lukáš D., Louda P., Mitura S. (2008). Potential applications of nanofiber textile covered by carbon coatings. J. Achiev. Mater. Manuf. Eng..

[6] El-Newehy M.H., Al-Deyab S.S., Kenawy E.-R., Abdel-Megeed A. (2011). Nanospider Technology for the Production of Nylon-6 Nanofibers for Biomedical Applications. J. Nanomater..

[7] Gopal R., Kaur S., Ma Z., Chan C., Ramakrishna S., Matsuura T. (2006). Electrospun Nanofibrous Filtration Membrane. J. Membr. Sci..

[8] Guo J., Zhang Q., Cai Z., Zhao K. (2016). Preparation and Dye Filtration Property of Electrospun Polyhydroxybutyrate–Calcium Alginate/Carbon Nanotubes Composite Nanofibrous Filtration Membrane. Sep. Purif. Technol..

[9] Homaeigohar S.S., Buhr K., Ebert K. (2010). Polyethersulfone Electrospun Nanofibrous Composite Membrane for Liquid Filtration. J. Membr. Sci..

[10] Zhu M., Han J., Wang F., Shao W., Xiong R., Zhang Q., Pan H., Yang Y., Samal S.K., Zhang F. (2016). Electrospun Nanofibers Membranes for Effective Air Filtration. Macromol. Mater. Eng..

[11] Bhattarai S.R., Bhattarai N., Yi H.K., Hwang P.H., Cha D.I., Kim H.Y. (2004). Novel Biodegradable Electrospun Membrane: Scaffold for Tissue Engineering. Biomaterials.

[12] Kim H.-W., Lee H.-H., Knowles J.C. (2006). Electrospinning Biomedical Nanocomposite Fibers of Hydroxyapatite/Poly(Lactic Acid) for Bone Regeneration. J. Biomed. Mater. Res. Part A.

[13] Lao L., Wang Y., Zhu Y., Zhang Y., Gao C. (2011). Poly(Lactide-Co-Glycolide)/Hydroxyapatite Nanofibrous Scaffolds Fabricated by Electrospinning for Bone Tissue Engineering. J. Mater. Sci. Mater. Med..

[14] Yoshimoto H., Shin Y.M., Terai H., Vacanti J.P. (2003). A Biodegradable Nanofiber Scaffold by Electrospinning and Its Potential for Bone Tissue Engineering. Biomaterials.

[15] Lannutti J., Reneker D., Ma T., Tomasko D., Farson D. (2007). Electrospinning for Tissue Engineering Scaffolds. Mater. Sci. Eng. C.

[16] Liang D., Hsiao B.S., Chu B. (2007). Functional Electrospun Nanofibrous Scaffolds for Biomedical Applications. Adv. Drug Deliv. Rev..

[17] Pourhojat F., Shariati S., Sohrabi M., Mahdavi H., Asadpour L. (2018). Preparation of antibacterial electrospun Poly lactic-co–glycolic acid nanofibers containing Hypericum Perforatum with bedsore healing property and evaluation of its drug release performance. Int. J. Nano Dimens..

[18] Khil M.-S., Cha D.-I., Kim H.-Y., Kim I.-S., Bhattarai N. (2003). Electrospun Nanofibrous Polyurethane Membrane as Wound Dressing. J. Biomed. Mater. Res..

[19] Chen J.-P., Chang G.-Y., Chen J.-K. (2008). Electrospun collagen/chitosan nanofibrous membrane as wound dressing. Colloids Surf. A Physicochem. Eng. Asp..

[20] Zahedi P., Rezaeian I., Ranaei-Siadat S.-O., Jafari S.-H., Supaphol P. (2010). A Review on Wound Dressings with an Emphasis on Electrospun Nanofibrous Polymeric Bandages. Polym. Adv. Technol..

[21] Sill T.J., von Recum H.A. (2008). Electrospinning: Applications in Drug Delivery and Tissue Engineering. Biomaterials.

[22] Zeng J., Xu X., Chen X., Liang Q., Bian X., Yang L., Jing X. (2003). Biodegradable Electrospun Fibers for Drug Delivery. J. Control. Release.

[23] Schnell E., Klinkhammer K., Balzer S., Brook G., Klee D., Dalton P., Mey J. (2007). Guidance of Glial Cell Migration and Axonal Growth on Electrospun Nanofibers of Poly-ε-Caprolactone and a Collagen/Poly-ε-Caprolactone Blend. Biomaterials.

[24] Jung H.-R., Ju D.-H., Lee W.-J., Zhang X., Kotek R. (2009). Electrospun Hydrophilic Fumed Silica/Polyacrylonitrile Nanofiber-Based Composite Electrolyte Membranes. Electrochim. Acta.

[25] Kim J., Reneker D.H. (1999). Mechanical Properties of Composites Using Ultrafine Electrospun Fibers. Polym. Compos..

[26] Brząkalski D., Przekop R.E., Dobrosielska M., Sztorch B., Marciniak P., Marciniec B. (2020). Highly Bulky Spherosilicates as Functional Additives for Polyethylene Processing—Influence on Mechanical and Thermal Properties. Polym. Compos..

[27] Voronkov M.G., Lavrent’yev V.I., Bosche F.L. (1982). Inorganic ring systems. Topics in Current Chemistry.

[28] Lickiss P.D., Cordess D.B., Hill A.F., Fink M.J. (2008). Fully Condensed Polyhedral Oligosilsesquioxanes (POSS): From synthesis to application. Advances in Organometallic Chemistry.

[29] Hartmann-Thompson C. (2011). Applications of polyhedral oligomeric silsesquioxanes. Advances in Silicon Science.

[30] Scott D.W. (1946). Thermal Rearrangement of Branched-Chain Methylpolysiloxanes. J. Am. Chem. Soc..

[31] Du Y., Liu H. (2020). Cage-like Silsesquioxanes-Based Hybrid Materials. Dalt. Trans..

[32] Tanaka K., Chujo Y. (2012). Advanced Functional Materials Based on Polyhedral Oligomeric Silsesquioxane (POSS). J. Mater. Chem..

[33] Marciniec B. (2009). Hydrosilylation.

[34] Cicala G., Blanco I., Latteri A., Ognibene G., Agatino Bottino F., Fragalà M. (2017). PES/POSS Soluble Veils as Advanced Modifiers for Multifunctional Fiber Reinforced Composites. Polymers.

[35] Deng N., Wang L., Liu Y., Zhong C., Kang W., Cheng B. (2020). Functionalized Polar Octa(γ-Chloropropyl) Polyhedral Oligomeric Silsesquioxane Assisted Polyimide Nanofiber Composite Membrane with Excellent Ionic Conductivity and Wetting Mechanical Strength towards Enhanced Lithium-Ion Battery. Compos. Sci. Technol..

[36] Zhang Q., Liu Y., Ma J., Zhang M., Ma X., Chen F. (2019). Preparation and Characterization of Polypropylene Supported Electrospun POSS-(C3H6Cl)8/PVDF Gel Polymer Electrolytes for Lithium-Ion Batteries. Colloids Surf. A Physicochem. Eng. Asp..

[37] Zhang M., Ma X., Liu Y., Ma J., Chen F., Zhang Q. (2018). High-Performance Electrospun POSS-(PMMA46)8/PVDF Hybrid Gel Polymer Electrolytes with PP Support for Li-Ion Batteries. Ionics.

[38] Zhao H., Deng N., Yan J., Kang W., Ju J., Wang L., Li Z., Cheng B. (2019). Effect of OctaphenylPolyhedral Oligomeric Silsesquioxane on the Electrospun Poly-m-Phenylene Isophthalamid Separators for Lithium-Ion Batteries with High Safety and Excellent Electrochemical Performance. Chem. Eng. J..

[39] Jeong H.-G., Han Y.-S., Jung K.-H., Kim Y.-J. (2019). Poly(Vinylidene Fluoride) Composite Nanofibers Containing Polyhedral Oligomeric Silsesquioxane–Epigallocatechin Gallate Conjugate for Bone Tissue Regeneration. Nanomaterials.

[40] Liu B., Yao T., Ren L., Zhao Y., Yuan X. (2018). Antibacterial PCL Electrospun Membranes Containing Synthetic Polypeptides for Biomedical Purposes. Colloids Surf. B Biointerfaces.

[41] Brząkalski D., Sztorch B., Frydrych M., Pakuła D., Dydek K., Kozera R., Boczkowska A., Marciniec B., Przekop R.E. (2020). Limonene Derivative of Spherosilicate as a Polylactide Modifier for Applications in 3D Printing Technology. Molecules.

[42] Ye M., Wu Y., Zhang W., Yang R. (2018). Synthesis of incompletely caged silsesquioxane (T7-POSS) compounds via a versatile three-step approach. Res. Chem. Intermed..

[43] Vieira E.G., Dal-Bó A.G., Frizon T.E.A., Dias Filho N.L. (2017). Synthesis of two new Mo(II) organometallic catalysts immobilized on POSS for application in olefin oxidation reactions. J. Organomet. Chem..

[44] Brząkalski D., Przekop R.E., Frydrych M., Pakuła D., Dobrosielska M., Sztorch B., Marciniec B. (2022). Where ppm Quantities of Silsesquioxanes Make a Difference—Silanes and Cage Siloxanes as TiO_2_ Dispersants and Stabilizers for Pigmented Epoxy Resins. Materials.

[45] Mofokeng J.P., Luyt A.S., Tábi T., Kovács J. (2011). Comparison of Injection Moulded, Natural Fibre-Reinforced Composites with PP and PLA as Matrices. J. Thermoplast. Compos. Mater..

[46] Naeem S., Gilani S.Q.Z., Baheti V., Wiener J., Militky J., Javed S., Ali A., Javed Z., ul Hassan S.Z. (2017). Electrical Conductivity of PLA Films Reinforced with Carbon Nano Particles from Waste Acrylic Fibers. Advances in Natural Fibre Composites.

[47] Brown J.F., Vogt L.H. (1965). The Polycondensation of Cyclohexylsilanetriol. J. Am. Chem. Soc..

[48] Spirk S., Nieger M., Belaj F., Pietschnig R. (2009). Formation and hydrogen bonding of a novel POSS-trisilanol. Dalton Trans..

[49] Ozdemir E., Lekesiz T.O., Hacaloglu J. (2016). Polylactide/Organically Modified Montmorillonite Composites; Effects of Organic Modifier on Thermal Characteristics. Polym. Degrad. Stab..

[50] Mayes A. (2005). Softer at the boundary. Nature Mater..

[51] Brząkalski D., Przekop R.E., Sztorch B., Jakubowska P., Jałbrzykowski M., Marciniec B. (2020). Silsesquioxane Derivatives as Functional Additives for Preparation of Polyethylene-Based Composites: A Case of Trisilanol Melt-Condensation. Polymers.

